# The Value of Microvascular Imaging for Triaging Indeterminate Cervical Lymph Nodes in Patients with Papillary Thyroid Carcinoma

**DOI:** 10.3390/cancers12102839

**Published:** 2020-10-01

**Authors:** Seongyong Lee, Ji Ye Lee, Ra Gyoung Yoon, Ji-hoon Kim, Hyun Sook Hong

**Affiliations:** 1 Department of Radiology, Eulji Medical Center, Eulji University College of Medicine, Seoul 01830, Korea; 20170141@eulji.ac.kr (S.L.); rgyoon@eulji.ac.kr (R.G.Y.); 2 Department of Radiology, Seoul National University Hospital, Seoul National University College of Medicine, Seoul 03080, Korea; jihnkim@snu.ac.kr; 3 Department of Radiology, Soonchunhyang University Bucheon Hospital, Soonchunhyang University College of Medicine, Bucheon 14584, Korea; hshong@schmc.ac.kr

**Keywords:** ultrasonography, Doppler, lymph node metastasis, thyroid cancer, biopsy

## Abstract

**Simple Summary:**

Papillary thyroid carcinomas (PTC) are indolent tumors associated with excellent long-term survival, albeit frequently accompanied by cervical lymph node (LN) metastasis. The imaging criteria using conventional ultrasound (US) techniques showed high diagnostic performance for the suspicious and probably benign LN categories, but showed low diagnostic performance for the indeterminate category. In this retrospective study, we aimed to assess the added value of Superb Microvascular Imaging (SMI) for detecting metastatic PTC in the indeterminate LN category. We confirmed that SMI could effectively stratify indeterminate LNs by visualizing additional vascular signals. The reclassified categories of SMI provided a high diagnostic performance to distinguish metastasis from benign LNs. Therefore, adding SMI to conventional US scans can be useful when evaluating indeterminate LNs in patients with PTC.

**Abstract:**

Assessment of lymph node (LN) status in patients with papillary thyroid carcinoma (PTC) is often troublesome because of cervical LNs with indeterminate US (ultrasound) features. We aimed to explore whether Superb Microvascular Imaging (SMI) could be helpful for distinguishing metastasis from indeterminate LNs when combined with power Doppler US (PDUS). From 353 consecutive patients with PTC, LNs characterized as indeterminate by PDUS were evaluated by SMI to distinguish them from metastasis. Indeterminate LNs were reclassified according to the SMI, the malignancy risk of each category was assessed, and the diagnostic performance of suspicious findings on SMI was calculated. The incidence of US-indeterminate LNs was 26.9%. Eighty PDUS-indeterminate LNs (39 proven as benign, 41 proven as malignant) were reclassified into probably benign (*n* = 26), indeterminate (*n* = 20), and suspicious (*n* = 34) categories according to SMI, with malignancy risks of 19.2%, 20.0%, and 94.1%, respectively. After combining SMI with PDUS, 80.8% (21/26) of probably benign LNs and 94.1% (32/34) of suspicious LNs could be correctly diagnosed as benign and metastatic, respectively. The diagnostic sensitivity, specificity, and accuracy of categorizing LNs as suspicious based on SMI were 78.1%, 94.9%, and 86.3%, respectively. In conclusion, the combination of SMI with PDUS was helpful for the accurate stratification of indeterminate LNs based on US in patients with PTC.

## 1. Introduction

Papillary thyroid cancers (PTC) are indolent tumors that are associated with excellent long-term survival. Although the mortality rate of patients with differentiated thyroid cancer has consistently been very low, up to 60–70% of patients have cervical lymph node (LN) metastasis at the time of surgery [1,2], which is currently regarded as an important risk factor for poor prognosis [3,4]. In PTC, it is assumed that tumor cells gain access to the lymphatic system either by the proliferation and invasion of new lymphatics into and around the developing tumor or by cells invading preexisting lymphatics adjacent to the tumor [5,6,7]. According to the current American Thyroid Association guideline, surgery is the treatment of choice for locoregional disease [8]. However, as wider neck dissections can increase the risk of surgical complications, accurate determination and mapping of cervical LN metastasis are important for patients with PTC. 

Ultrasonography (US) is the imaging method of choice for detecting and characterizing cervical LNs in patients with thyroid cancer [9,10,11,12,13]. According to the guidelines of the European Thyroid Association and the Korean Society of Thyroid Radiology, cervical LNs in patients with PTC are categorized into three groups according to US features: suspicious, indeterminate, and benign [11]. Increased abnormal vascularity in the LN is a well-known imaging feature of metastatic PTC that has been widely utilized [11,13]. Indeterminate LNs are defined as LNs with no imaging findings of suspicious or benign LNs [13]. A recently published study showed the frequent occurrence of US-indeterminate LNs (23.6% among biopsied LNs in thyroid cancer patients), and demonstrated that the indeterminate category had a higher malignancy risk (19.5%) than the probably benign category (2.8%) [14]. Suspicious US features on conventional US showed high specificity and predictivity for diagnosing metastatic PTC [13]. However, the proper management of LNs with US-indeterminate features still remains elusive. Although a recent study based on computed tomography (CT) analysis had addressed this issue [15], no breakthrough trials using US have been reported for the better differentiation of metastasis from indeterminate LNs. 

Power Doppler US (PDUS) has been accepted as a quick and noninvasive method for assessing vessels in tumor tissues. Specifically, along with gray-scale US, Doppler US has been adopted as a useful technique because of its ability to detect the hypervascular characteristic of metastatic PTC relative to a hypovascular normal lymphoid tissue background [11,13]. However, it is not possible to differentiate low-velocity flow from artifacts caused by background tissue motion on PDUS. A recently developed Doppler technique, known as Superb Microvascular Imaging (SMI; Canon Medical System, Otawara, Japan), can improve the visualization of small and low-velocity flow blood vessels by using an advanced Doppler algorithm. This algorithm separates low-velocity flow signals from clutter artifacts and provides high sensitivity for the visualization of low-velocity flow with high resolution [16]. Investigators have reported that SMI can detect more flow signals within tumors than conventional color Doppler or PDUS and thereby complement conventional Doppler scans in the examination of tumorous lesions [17,18,19,20].

A few studies have reported that SMI could be useful for improving the diagnostic performance during the assessment of cervical LNs [21,22,23] and salivary gland tumors [24]. However, its clinical utility for the prediction of PTC metastasis in LNs has not yet been validated. Considering the technical strengths of SMI, we hypothesized that the vascular information obtained by SMI could enhance the differentiation of metastatic and benign LNs in patients with PTC. Therefore, we investigated whether a combination of SMI and PDUS could be helpful for assessing US-indeterminate LNs in patients with PTC.

## 2. Results

### 2.1. Demographic Data

The incidence of indeterminate LNs in patients with PTC was 26.9%. Among 80 patients with indeterminate LNs, 39 patients had benign LNs, and 41 patients had metastatic LNs. 

Table 1 summarizes the demographic data of the patients and the characteristics of the indeterminate LNs. Patients who had metastatic LNs were significantly older than those who had benign LNs (*p* = 0.001). There were no significant differences in the sex, surgical treatment status, tumor extent, and BRAF^V600E^ mutation status between the two groups. 

The location of the LNs was significantly different (*p* < 0.001) between the metastatic and benign groups: level 2 was the most common location of LNs in the benign group, and level 4 was the most common location of LNs in the metastatic group. 

### 2.2. Analysis of Vascular Patterns on SMI

**Figure 1:**
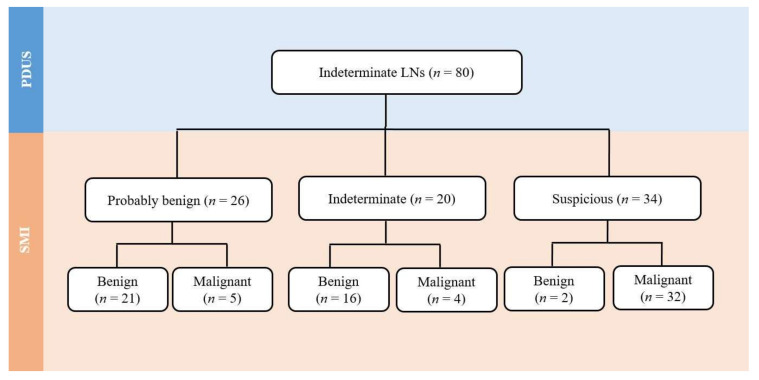
Reclassification of PDUS indeterminate LNs after the addition of SMI. LN—Lymph node, PDUS—Power Doppler ultrasound, SMI—Superb Microvascular Imaging.

**Figure 2:**
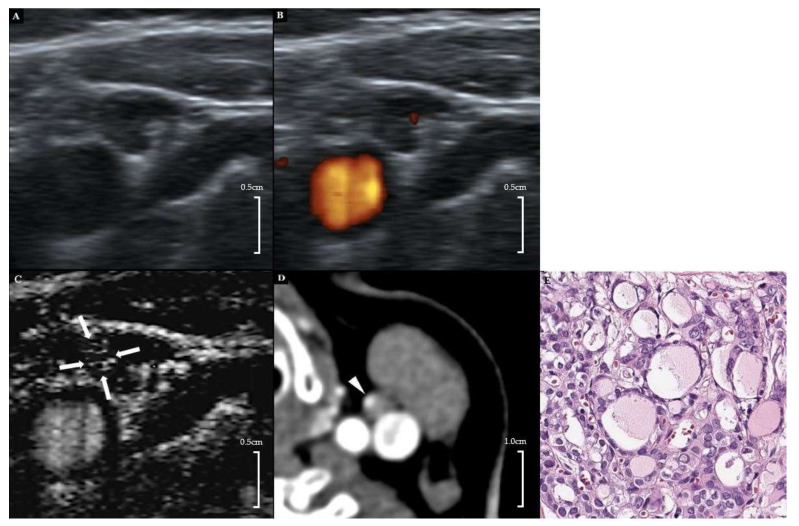
A 42-year-old female patient with left PTC. (**A**) Gray-scale US showed an ovoid hypoechoic indeterminate LN with displaced hilar echogenicity in the left neck level 3. (**B**) On PDUS, a displaced hilar vascularity was noted. (**C**) On SMI, an additional peripheral vascular signal was noted in the medial peripheral portion of the LN (arrows). (**D**) Contrast-enhanced CT (early arterial phase, 25 s) showed focal cortical contrast enhancement in the corresponding area (arrowhead). (**E**) Subsequent FNA and modified radical neck dissection revealed a metastatic papillary thyroid carcinoma (H&E stain, ×200). PTC-papillary thyroid carcinoma, US—ultrasound, LN—lymph node, PDUS—power Doppler ultrasound, SMI—superb microvascular imaging, FNA—fine needle aspiration.

**Figure 3:**
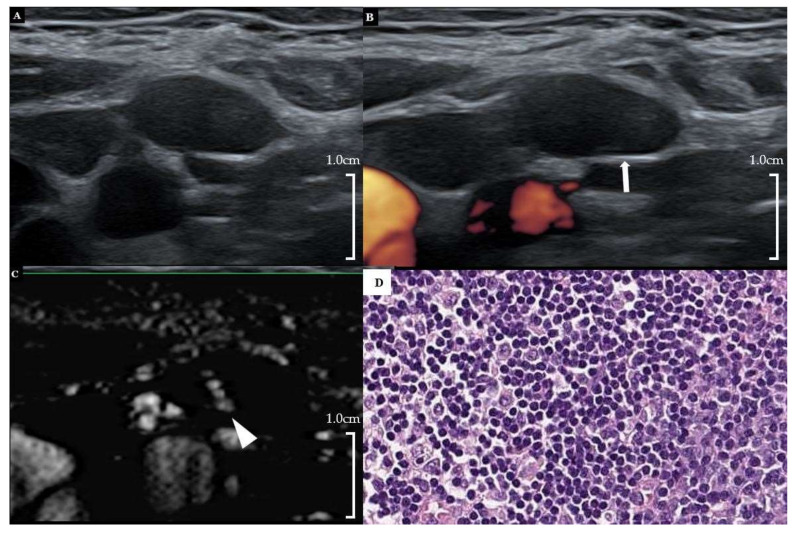
A 30-year-old female patient with right PTC. (**A**) Gray-scale US showed an ovoid hypoechoic indeterminate LN with loss of hilar echogenicity at right neck level 2. (**B**) Hilar vascularity was also absent on PDUS (arrow). (**C**) On SMI, hilar vascularity was noted in the central area of the LN. (**D**) Subsequent FNA and surgical biopsy revealed reactive hyperplasia (FNA-thyroglobulin = 0.16 ng/mL) (H&E stain, ×200). PTC = papillary thyroid carcinoma, US = ultrasound, LN = lymph node, PDUS = power Doppler ultrasound, SMI = superb microvascular imaging, FNA = fine needle aspiration.

The use of SMI revealed that 26 out of 80 LNs (32.5%) showed a centrally located vascular hilum without aberrant vascular signals. However, 22 out of 80 LNs (27.5%) revealed peripheral vascular signals, and 12 out of 80 LNs (15.0%) showed both central and peripheral vascular signals on SMI. On the basis of the SMI images, 42.5% (34 out of 80) were reclassified as suspicious LNs by showing additional peripheral vascular signals, and 32.5% (26 out of 80) were reclassified as probably benign because of central hilar vascular signals; however, 25.0% (20 out of 80) still remained in the indeterminate category. Figure 1 shows the flow chart depicting the changes of the LN categories after the addition of SMI to the use of PDUS. Figure 2 and Figure 3 illustrate representative cases. All patients with metastatic LNs showed BRAF^V600E^ mutations in the primary tumor. However, there were no cases of any aggressive subtypes of PTC.

Table 2 lists the incidence and malignancy risk of each LN category on SMI. Based on SMI characterization, the malignancy risks of suspicious LNs were significantly higher than those of the indeterminate and probably benign LN groups (*p* < 0.001), whereas the malignancy risks of indeterminate LNs were similar to those of the probably benign group (*p* = 0.81).

### 2.3. Diagnostic Performance of SMI

Of the 26 LNs reclassified as probably benign, 21 cases (80.7%) were correct. Of the 34 LNs reclassified as suspicious, 32 cases (94.1%) were correctly diagnosed as being malignant. Four LNs in the SMI indeterminate category were confirmed to be malignant. The sensitivity, specificity, positive predictive value, negative predictive value, and accuracy of SMI were 78.1%, 94.9%, 94.1%, 80.4%, and 86.3%, respectively. Table 3 summarizes the diagnostic performance of suspicious SMI features in differentiating metastatic and benign LNs.

### 2.4. Inter-Observer Agreement

The inter-observer agreement was moderate (κ = 0.79 [95% confidence interval [CI], 0.68‒0.92]) for the reclassified SMI categories of probably benign, indeterminate, and suspicious LNs. Agreement between the presence of central hilum (κ = 0.85 [95% CI, 0.72–0.98]) and peripheral vascular signals (κ = 0.83 [95% CI, 0.70–0.96]) was strong. 

## 3. Discussion

In this study, adding SMI to PDUS in the indeterminate LNs allowed the efficient differentiation of metastatic and benign LNs for patients with PTC by allowing better visualization of minute vascular signals and subsequently reducing the proportion of the indeterminate LN category. Although several studies have focused on the feasibility of SMI for evaluating cervical LNs, this is the first study to evaluate the value of SMI for distinguishing metastatic PTC from the specific population of US-indeterminate LNs. Most of the metastatic LNs from PTC are pathologically hypervascular when compared to benign lymphoid tissue [25,26], and abnormal vascularity on PDUS is a well-known imaging feature for diagnosing metastatic LNs in thyroid cancer. Hypervascularity in metastatic LNs is associated with increased tumor perfusion related to tumor angiogenesis and recruitment of capsular vessels [27,28]. In thyroid cancer, the US criteria for suspicious LNs (cystic change, calcification, hyperechogenicity, and abnormal vascularity) have been reported to be highly predictive (80–90%) of LN metastases [29,30]. For cervical LNs, the presence of a normal hilum is generally regarded as benign, and the absence of echogenic hilum or hilar vascularity is regarded as pathologic, because this may reflect the interruption of lymphatic flow by tumor invasion [27]. However, the hilum is observed in 28.6–87% of normal LNs on gray-scale US, and normal vascularization is observed in approximately two-thirds of normal LNs on Doppler scans [1,29,31,32]. Accordingly, the specificity for hilum loss has been reported to be only 29% for predicting the presence of metastasis [29,33]. In contrast, various benign conditions occurring along with LNs and persistent inflammatory stimuli can cause unusual morphological changes in the nodal shape in the neck, and this can result in a false positive presentation of US-indeterminate LNs with deformed or displaced hilum [27]. For this reason, indeterminate LNs are often encountered during routine practice [14], and this frequent manifestation of indeterminate LNs limits the accuracy of US, even with the application of the size criteria [11,13,14]. 

In our study, patients with metastatic LNs were older than those with benign LNs. Our results contradict previous reports, which consistently reported the aggressive nature of tumors in young thyroid cancer patients [34,35]. This might be attributed to the presence of prominent lymphoid tissues in young patients, which might occasionally appear as indeterminate LNs on US [36]. Likewise, the high prevalence of level 2, PDUS-indeterminate LNs among benign LNs might be associated with a similar reason because LNs in the upper jugular stations could easily be affected by benign hyperplasia due to various inflammatory diseases of the head and neck region [36].

Our results are consistent with those of previous studies, which demonstrated the effectiveness of SMI for differentiating malignant and benign cervical LNs [22,23]. In our study, many benign LNs with indeterminate US features exhibited additional central hilar vessels in SMI, and this may be attributed to the improved visualization of central hilar vessels with low-velocity flow. On conventional Doppler, a relatively low number of backscattering red blood cells in the small vessels could decrease the Doppler signal intensity, which may not surpass the noise level and may be overlooked. Moreover, low-velocity flow signals used in PDUS may be suppressed by a high-level single-wall filter. The use of SMI allowed further visualization of peripheral vessels in the metastatic LNs and improved visualization of aberrant tumor vessels with low-velocity flow. When metastatic tumors affect LNs, tumors entering the LNs usually progress in a centripetal fashion and exhibit peripheral vascularity with preserved central hilar vascularity. When the peripheral tumor nest is too small to be visualized on conventional scans, the hyperechogenicity or hypervascularity of the tumor can be overlooked. In those instances, SMI could enhance the accuracy of the diagnosis of metastatic LNs by allowing the detection of minute neovascular signals.

In our study, the incidence of indeterminate LNs was much reduced for SMI, and many of those indeterminate LNs were either classified as probably benign or suspicious. Moreover, the malignancy risks of indeterminate and probably benign LNs were similar in the categories reclassified on the basis of SMI. Based on these results, indeterminate LNs could be stratified into two groups of ‘suspicious’ vs. ‘indeterminate/probably benign’ on SMI. The diagnostic performance of suspicious findings on SMI for PDUS–indeterminate LNs was high, even similar to the reported diagnostic performance of suspicious findings on conventional gray–scale and Doppler scans [29,30]. 

The correct identification of probably benign LNs could potentially decrease the numbers of unnecessary biopsies and neck dissections, whereas the correct diagnosis of suspicious LNs could enhance accurate preoperative surgical mapping and eventually reduce the rate of recurrent or persistent disease (4). In the postoperative setting, an accurate triage of imaging-detected neck lesions can contribute to a more tailored postoperative risk stratification, which will enable the clinician to establish the management plan for patients with greater confidence. Our results highlight the value of combining SMI with PDUS for the accurate classification of indeterminate LNs. SMI could serve as a complementary imaging technique to standard US examinations, which can help refine candidates for LN biopsy in thyroid cancer patients without any concerns of ionizing radiation or intravenous contrast administration in CT.

Our study has several limitations. First, this was a retrospective study in which selection bias is inevitable with a small sample size. There were two reasons for this small sample size. First, SMI is a relatively new US technique because of which the time period was not long enough to validate this technique. Second, we attempted to include patients with a complete dataset of video clips (which requires time and effort when evaluating numerous LNs in routine practice) along with cytopathological verification. In practice, LNs with a typical benign appearance are rarely confirmed cytopathologically. Because we aimed to perform a node-by-node correlation for the determination of the precise accuracy of this technique, we focused on indeterminate LNs for which an unnecessary biopsy is frequently performed. Despite the disadvantages of our small subject number, we believe that the coherent results of our study reflect the validity of this relatively new technique. The second limitation is that retrospective assessment of US images inherently limits the accuracy of interpretation and detection of small vascular signals. Nevertheless, repeated inspections of the video clips might have mitigated the drawbacks of this retrospective evaluation. Further studies with a multicenter prospective setting and larger sample size are needed to validate our results. The third limitation is that the additional vascular signals detected on SMI may be either microscopic or macroscopic metastases. As guidelines [8,13] have suggested that microscopic nodal positivity does not carry the risk of recurrence of macroscopic (clinically detectable) disease, the exact clinical significance of small and low-velocity flow blood vessels in metastatic LNs in terms of patient outcome remains unclear. Further studies with histopathological investigation and longer follow-up are necessary. 

## 4. Materials and Methods

### 4.1. Patient Population

This retrospective study was conducted in accordance with the Declaration of Helsinki and approved by the Institutional Review Board of Soonchunhyang University Bucheon Hospital; the requirement for written informed consent was waived because of its retrospective nature. Among the 353 patients with primary (*n* = 219) or recurrent (*n* = 134) PTC who underwent US between December 2016 and December 2018, 95 patients showed 95 indeterminate LNs on conventional gray-scale and PDUS. Indeterminate LNs were defined as LNs without the imaging features of benign (LNs with fatty hilum or central hilar vascularity) or suspicious LNs (LNs with hyperechogenicity, cystic change, abnormal vascularity, or calcifications). The features of indeterminate LNs included an eccentric or deformed configuration of hilar vessels as well as the loss of central hilar vascularity on US, regardless of the nodal shape [13]. A total of 15 patients with a poor sonic window or prominent motion artifacts were excluded from the study. Finally, 80 patients (70 primary and 10 recurrent) with 80 indeterminate LNs were included in the study. 

### 4.2. US Examination

Using a high-resolution US unit equipped with a 14 MHz, high-frequency linear transducer (Aplio 500; Canon Medical Systems), two dedicated head and neck radiologists (H.S.H. and J.Y.L. with 27 and 7 years of experience in thyroid US and interventional procedures, respectively) performed US examinations, including gray-scale US, PDUS, and SMI, to assess indeterminate LNs based on gray-scale US and PDUS. In case of any indeterminate LN, they evaluated vascular patterns after applying SMI.

PDUS was performed with standardized parameters adjusted for high sensitivity with a low wall filter to allow the detection of blood vessels with low-velocity flow (scale, 4.9–6.1 cm/s; frame rate, 7–9 frames/s; pulse repetition frequency, 13.7–15.6 kHz). The gain was initially increased to show color noise and then decreased to avoid substantial artifacts. SMI was performed in the monochromatic mode, and the settings were standardized according to the manufacturer’s recommendations for a high frame rate combined with minimal flash artifacts (frame rate, 25–30 frames/s; pulse repetition frequency, 15.4–20.2 kHz) and a low velocity range (< 2 cm/s). The monochromatic mode was chosen because of its high sensitivity for detecting low-velocity flow and small blood vessels. Still images and video clips of the target LNs from PDUS and SMI were stored and archived on a picture archiving and communication system. 

### 4.3. Imaging Analysis

Two head and neck radiologists (J.Y.L. and H.S.H.) retrospectively evaluated the images and video clips of SMI independently and were blinded to the patients’ information, especially with regard to whether SMI depicted normal hilar vascularity, abnormal/peripheral/chaotic vascularity, or both. 

**Figure 4:**
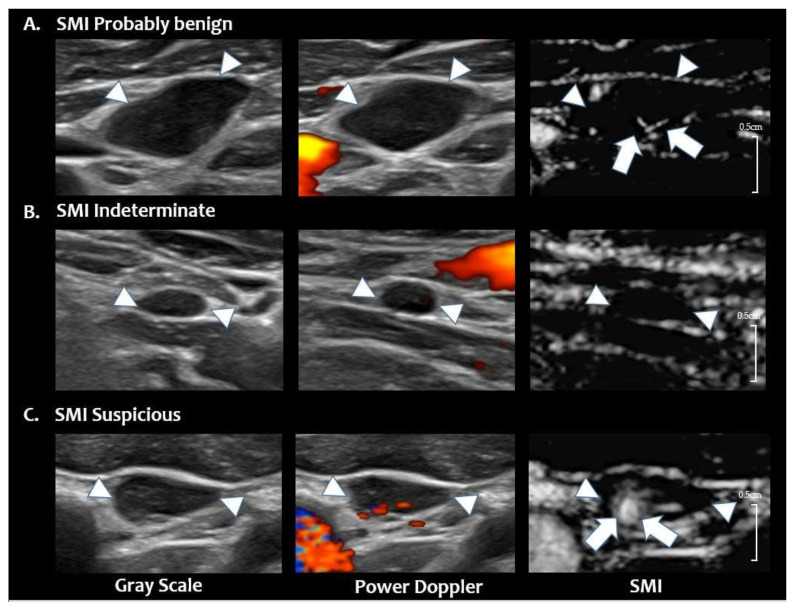
Reclassified LN categories of SMI. (**A**) SMI probably benign LN. Grey scale and PDUS images show a hypoechoic LN with loss of hilum and hilar vascularity in the left neck level 3. On SMI, central hilar vascularity is noted (arrows), and this LN is classified as ‘SMI probably benign’ (final diagnosis-benign on FNA-Tg) (**B**) SMI indeterminate LN. Grey scale and PDUS show a small LN in right neck level 4 with loss of hilum and hilar vascularity. On SMI, no additional vascular signals are noted; therefore, this LN is classified as ‘SMI indeterminate’ (final diagnosis-benign on FNA-Tg). (**C**) SMI suspicious LN. Grey scale and PDUS show a hypoechoic LN with eccentric cortical thickening and displaced hilar vascularity in left neck level 3. On SMI, abnormal peripheral vascularity is noted at the medial portion of the LN (arrows, final diagnosis-metastatic PTC on CNB). The arrowheads in figures A, B, C indicates the margin of the LN. PTC = papillary thyroid carcinoma, US = ultrasound, LN = lymph node, PDUS = power Doppler ultrasound, SMI = superb microvascular imaging, FNA = fine needle aspiration, CNB = core needle biopsy, Tg = thyroglobulin.

After independent analysis, consensus reading was performed for discordant cases. The LNs were re-categorized as probably benign, indeterminate, or suspicious LNs after the interpretation session of SMI. The LNs were categorized as ‘suspicious’ when they showed peripheral or abnormal diffuse vascularity on SMI. Probably benign LNs were diagnosed when non-displaced central hilar vascularity was evident without the presence of peripheral vascularity. The LNs with no imaging features of suspicious or probably benign LNs remained in the indeterminate category. Figure 4 shows representative cases of categories reclassified by SMI.

### 4.4. Reference Standard

For all indeterminate LNs, US-guided fine-needle aspiration (FNA) or core-needle biopsy (CNB) was performed by one of the two radiologists. Materials obtained from FNA were smeared, and the remaining aspirated samples were rinsed with 1 mL of isotonic saline and submitted for thyroglobulin measurement (FNA-Tg). The cut-off value of FNA-Tg for differentiating metastasis from benign lesions was 1 ng/mL [37] in this study. CNB was performed by targeting the LNs with a disposable, automatic, 18-gauge core-biopsy needle (TSK, Ace-Cut; Create Medic, Yokohama, Japan). All LNs that were diagnosed as metastasis on FNA/CNB were surgically confirmed through modified radical neck dissection (*n* = 65) or preoperative tattooing, followed by selective neck dissection (*n* = 15). The BRAF mutation was identified from a portion of the BRAF^V600E^ gene by real-time PCR using a detection kit (PNA Clamp BRAF Mutation Detection Kit, Panagene, Daejeon, Korea).

### 4.5. Statistical Analysis

Student’s *t*-test or Mann–Whitney U-test was used to analyze continuous variables of age as well as diameters of the primary tumor and LNs. $\chi^{2}$ test or Fisher’s exact test was used to analyze categorical variables to compare the demographic data (sex, treatment status, tumor multifocality, extrathyroidal extension, BRAF mutation status) and the characteristics of benign and metastatic LNs (LN laterality and location).

Cohen κ values were used to analyze the inter-observer agreement of the presence of a central hilum and abnormal vascularity and reclassify categories of LNs based on SMI. With SMI, incidence and malignancy risks were calculated for each diagnostic category and compared using a Fisher’s exact test. The diagnostic performance of suspicious findings on SMI for differentiating benign and metastatic LNs was assessed by calculating the sensitivity, specificity, accuracy, and positive and negative predictive values. All statistical analyses were performed with MedCalc version 18.6 statistical software (MedCalc Software bvba, Ostend, Belgium). The significance threshold for the differences was *p* < 0.05.

## 5. Conclusions

In conclusion, SMI was helpful for the accurate stratification of PDUS–indeterminate LNs in patients with PTC, thus reducing the frequency of US–indeterminate LNs in patients with PTC. The addition of SMI to the routine evaluation of indeterminate LNs could enhance accurate patient management in PTC and enable the recommendation of invasive procedures with greater confidence.

## Figures and Tables

**Table 1:** Demographic data of patients and characteristics of indeterminate LNs according to pathology.

Variables	Benign (*n* = 39)	Metastasis (*n* = 41)	*p*
Female patients	27 (69.2)	27 (65.9)	0.933
Age (mean)	43.4 ± 16.1	57.3 ± 20.2	0.001
Treatment status			
1st thyroid cancer surgery	34 (87.2)	36 (87.8)	0.824
Repeated thyroid cancer surgery	5 (12.8)	5 (12.2)	
Primary tumor			
Largest diameter, range (mm)	7.1–38.0	6.7–45.0	0.421
Multifocal tumor	22 (56.4)	26 (63.4)	0.586
Gross extrathyroidal extension			0.877
T3	2 (5.1)	4 (9.8)	
T4	0 (0)	1 (2.4)	
BRAF^V600E^ mutation	30 (76.9)	35 (85.4)	0.641
Mean minimal axial diameter of LN (mm)	8.1 ± 6.2	9.2 ± 5.9	0.533
Laterality of LN			0.877
Left	17 (43.5)	18 (44.0)	
Right	22 (56.4)	23 (56.1)	
Location of LN			0.001
1	1 (2.6)	0 (0.0)	
2	17 (43.6)	3 (7.3)	
3	6 (15.4)	10 (24.4)	
4	7 (17.9)	22 (53.6)	
5	4 (10.3)	0 (0.0)	
6	4 (10.3)	6 (14.6)	

Numbers in parentheses are percentages of patients in each group. LN—Lymph node.

**Table 2:** Malignancy risk of LNs categorized by SMI.

Category	Numbers (%)	Malignancy Risk (%)
Probably benign	26 (32.5)	5/26 (19.2)
Indeterminate	20 (25.0)	4/20 (20.0)
Suspicious	34 (42.5)	32/34 (94.1)

Numbers in parentheses are percentages in each group.

**Table 3:** Diagnostic performances of suspicious findings on SMI for detecting metastasis in the indeterminate LN category on conventional grey scale and Doppler US (*n* = 80).

Parameter	SMI
Sensitivity	78.1% (32/41)
Specificity	94.9% (37/39)
False-positive rate	5.1% (2/39)
False-negative rate	22.0% (9/41)
Accuracy	86.3 % (74/80)
Positive predictive value	94.1% (32/34)
Negative predictive value	80.4% (37/46)
